# Low-resistive vibratory penetration in granular media

**DOI:** 10.1371/journal.pone.0175412

**Published:** 2017-04-18

**Authors:** Baptiste Darbois Texier, Alejandro Ibarra, Francisco Melo

**Affiliations:** Departamento de Física Universidad de Santiago de Chile, Avenida Ecuador 3493, 9170124 Estación Central, Santiago, Chile; University of Michigan, UNITED STATES

## Abstract

Non-cohesive materials such as sand, dry snow or cereals are encountered in various common circumstances, from everyday situations to industry. The process of digging into these materials remains a challenge to most animals and machines. Within the animal kingdom, different strategies are employed to overcome this issue, including excavation methods used by ants, the two-anchor strategy employed by soft burrowers such as razor-clams, and undulatory motions exhibited by sandfish lizards. Despite the development of technology to mimic these techniques in diggers and robots, the limitations of animals and machines may differ, and mimicry of natural processes is not necessarily the most efficient technological strategy. This study presents evidence that the resisting force for the penetration of an intruder into a dry granular media can be reduced by one order of magnitude with small amplitude (*A* ≃ 10 *μ*m) and low frequency (*f* = 50 − 200 Hz) mechanical vibrations. This observed result is attributed to the local fluidization of the granular bed which induces the rupture of force chains. The drop in resistive force on entering dry granular materials may be relevant in technological development in order to increase the efficiency of diggers and robots.

## Introduction

The intrusion of a solid finger into a granular media is used as a model for studying complex root grow mechanics [1] and soil penetration tests [2]. Under these situations, there are still important unresolved questions and gaps in understanding on the effect of intruder geometry and granular properties on resistive force. A few animals have developed digging strategies through natural selection and evolutionary processes to overcome difficulties of digging into granular materials; however it still remains a challenge [3]. For instance, it was recently reported that the head oscillation of the Ocellated skink could reduce granular resistance force during burial locomotion, with the efficiency depending on the extent of the material’s cohesion [4]. The progressive improvements in understanding of these mechanisms in nature has inspired technology and design in the engineering of diggers and robots [5].

A rigid intruder is able to penetrate non-cohesive granular materials by producing a surrounding flow similar to the processes that occur in fluids. However, the quasi-static immersion of an intruder into a dry granular assembly requires a force that is several orders of magnitude larger than necessary in fluids under similar conditions [6–8]. This occurs as a result of the progressive formation of a network composed of force chains, which simultaneously increase in size with intruder penetration. In two dimensional geometries, such a network is limited by two symmetric shear bands, increasing in amplitude, that nucleate from near the fingertip, reach the free surface, and exhibit original dynamics with nucleation-relaxation processes as the indenter is buried, such as the finger progress [9]. In the case of three dimensions, intruder progression gives rise to a surface that is almost symmetrical to the axis, which initiates from the fingertip and concentrates the shear strain. According to the pioneering work of Hill *et al.* [6] the force experienced by the indenter of radius *R* at a penetration distance *z* is a power law of the ratio *z*/*R*, which is mainly attributed to the high pressure exerted by the tip on the granular sample. It is apparent that this scaling still lacks accurate understanding, due to the difficulties encountered in modeling stress distribution in compressed granular media and the eventual formation of shear bands. In particular, the fact that the previous law does not depend on the size of the grain remains undetermined.

There is a long history of studies investigating the effect of mechanical vibrations on granular media, driven by the observation of coherent structures in these media [10–12]. A considerable effort has been instigated through the initial observation of non-intuitive convection cells [13, 14], leading to size segregation [15, 16], and pattern formation [13, 17–19]. Moreover, highly vibrated granular media can reach a gas state and thus present analogies with phase transition phenomenon [20–22].

In addition to relatively large amplitude vibrations, it is necessary to consider the effect of small perturbations and sound propagation on the mechanical resistance of granular compacts. Acoustic and ultrasonic waves may induce local contact sliding as well as a significant decrease in material viscosity and bulk modulus weakening, even at a packed granular state [23]. This effect, referred to as acoustic fluidization, was first described in a geophysical context [24], and initially proposed as being responsible for the weakening of faults and triggering avalanches. Enlightening information on the structural changes induced in the material by acoustic fluidization has been recently obtained through sound diffusion and coda wave interferometry [25, 26].

Previous investigations are focused on how vibrations impact an entire area of granular materials, however there has been little consideration to cases where forces are applied locally and directly on the penetrating object. This study reports on the resistive force experienced by a vibrating cylindric intruder entering into a motionless and dry granular media. Results indicate that the resistive force is considerably reduced, up to ten times, due to the rupture of force chains caused by mechanical vibrations from the intruder inflicted at the surface. As the penetration deepens, the intruder decreases the vibration amplitude due to increasing resistance from the granular media. Subsequently, the effect of force reduction ceases partially at a critical depth, demonstrated as a non-trivial function of the free acceleration of the object. Observation of granular flow in a reduced two dimensional geometry has revealed that vibrations induce the development of a zone of convecting grains, dramatically reducing the effect of shear bands and as a result, friction. As penetration exceeds the critical depth the convecting zone is positioned close to the free surface of the media. A scaling for the size of this zone is proposed.

## Materials and methods

**Fig 1:**
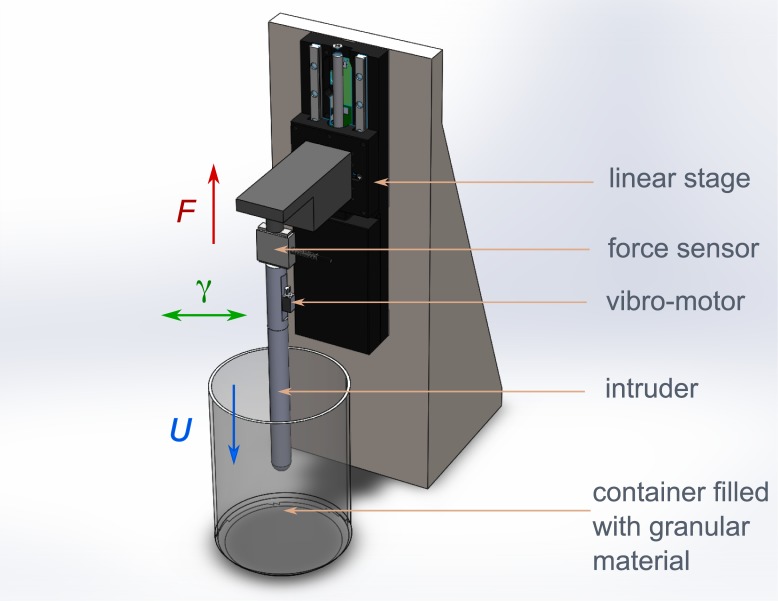
Sketch of the experimental setup and parameters.

In order to study the vertical penetration of a rigid intruder into a granular medium, the setup follows the diagram in Fig 1. The intruder is an acrylic cylinder, mounted on a motorized translational stage (Thorlabs MTS-50) moving vertically at a constant velocity *U* between 0.1 and 2.5 mm/s. The intruder diameter 2*R* varies between 6 and 12 mm, and the tip varies between a rounded and conical shape with an angle *ϕ* varying from 15 to 180° (corresponding to a flat tip). Two different granular media are considered, both are monodisperse glass spheres of bulk density *ρ* = 2.46 × 10^3^ kg/m^3^ but different in diameter: *d* = (250 ± 25) *μ*m and *d* = (1.0 ± 0.1) mm. The granular media is confined into a cylindrical tank of 15 cm in diameter and 20 cm in depth. These dimensions are sufficient so that coupling of the granular flow with container walls is prevented [27]. The initial compaction *φ* of the media is controlled by gently tapping the container several times before each experiment. This procedure is acknowledged as a technique to set the system at the random close-packing volume fraction [28, 29]. All experiments are done at constant air humidity *R*_*H*_ ≃ (37 ± 2)% and temperature *T* ≃ 20.0°C, therefore it is possible to neglect potential cohesion between grains. A force sensor (Futek LBS200 S-Beam) is used to measure the vertical resisting force imposed upon the intruder, operating within the range of ±2 N, with an absolute error of 3 mN. In addition, an eccentric motor (Precision Microdrive 304-008 Picovibe) with voltage control is clamped on top of the intruder. This motor will only impose lateral vibrations to the intruder, independently measured with an accelerometer (PCB Piezoelectronics 352A24). Lateral vibrations of the intruder are characterized when the intruder is not immersed in the granular media. Despite the elongated geometry of the intruder, only harmonic vibrations are observed along the surface of the intruder, with a near constant acceleration amplitude *γ*, within 7% accuracy. Moreover, a modification in the position or the orientation of the vibro-motor along the intruder does not induce significant changes in the acceleration of vibrations.

## Experimental results

**Fig 2:**
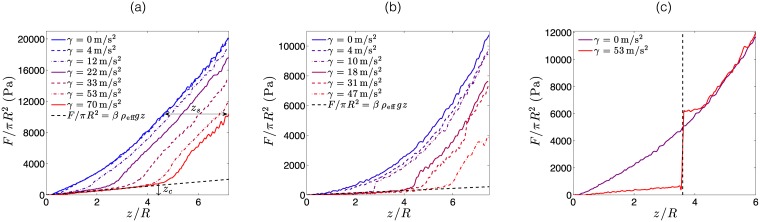
Resistive pressure *F*/*πR*^2^ as a function of the normalized penetration depth *z*/*R* for a vertical penetration of a cylindric intruder under various vibration accelerations *γ*: (a) Cylinder of radius *R* = 5 mm with a rounded tip; (b) Cylinder of radius *R* = 5 mm with a conical tip of angle *ϕ* = 15°. All the experiments were performed in a granular media made of glass beads of diameter *d* = 250 *μ*m. The dark dashed lines indicate the expected force for a quasi-static entry of the intruder into a fluidized bed, of effective density *ρ*_eff_ = *ρφ* and numerical coefficients *β* = 2.6 for (a) and *β* = 1.1 for (b). In (a), the arrows indicate the critical depth *z*_*c*_ and the vertical shift *z*_*s*_ for an acceleration *γ* = 70m/s^2^ (red solid line). (c) Comparison of the resistive pressure without acceleration vibrations (purple line) and with acceleration vibrations *γ* = 53m/s^2^ (red line), that cease at the depth denoted by the dark dashed line. Such experiments are created with a cylinder of 4 mm in radius with a rounded tip, penetrating into a granular bed made of glass beads of 250 *μ*m in diameter.

In absence of vibrations (*γ* = 0m/s^2^), the intruder experiences a force *F* which is a power law of the depth immersion *z* as shown in Fig 2a and 2b and confirming the observations of Hill *et al.* [6]. This study also reports the effect of the intruder’s tip geometry on the resisting force, as observed in the present experiments. Furthermore, according to negligible edge effects, an increase in the size or change in the shape of the container does not induce any modification of the experimental results. Moreover, the penetrating velocity has no effect on the force-depth profile within the range of this experimental setup. This result implies that these experiments take place in a quasi-static regime, consistent with the conclusions of Albert *et al.* [30].

**Fig 3:**
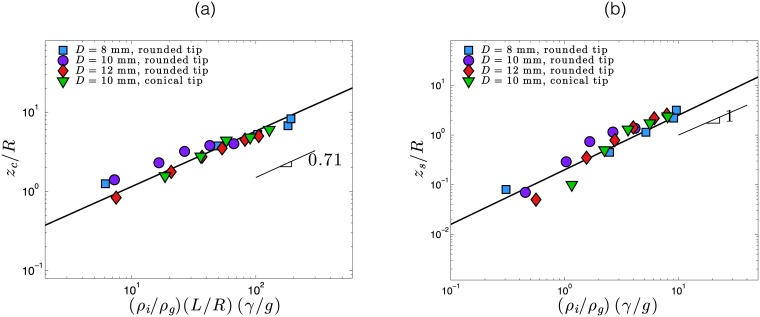
(a) The measured critical normalized depth *z*_*c*_/*R* as a function of the experimental parameter (*ρ*_*i*_/*ρ*_*g*_)(*L*/*R*)(*γ*/*g*). *z*_*c*_ is defined as the depth where the relative difference is maximized (*F*_*γ*_ − *F*_*γ* = 0_)/*F*_*γ* = 0_. The black solid line is the best fit line for the data with a power law of exponent 0.71. (b) Measured shifted normalized depth *z*_*s*_/*R* as a function of the experimental parameter (*ρ*_*i*_/*ρ*_*g*_)(*γ*/*g*). *z*_*s*_ is defined as the required shift to superimpose the curve force on the reference without vibrations in the range *z* > *z*_*c*_. In the two plots, blue squares, purple dots and red diamonds correspond to a cylindrical intruder of 8, 10 and 12 mm in diameter, respectively, with a rounded tip. Green triangles correspond to a cylindrical intruder of 10 mm in diameter with a conical tip of angle *ϕ* = 15°.

The effect of mechanical vibrations on the force experienced by the intruder is investigated. Fig 2a and 2b show the resistive pressure on the intruder from mechanical vibrations at different acceleration intensities. It is observed that during penetration into the granular media, vibrations decrease the resistive force on the intruder. This effect was observed under all variations of intruder geometry, and in all the granular materials tested in our experiments. It is noted that *F* is lowered up to a factor ten by the presence of vibrations (as reported in Fig 2b) with a conical tip and *γ* = 47m/s^2^. The main result of this study is the observation of a considerable drop in the resistive force induced by small amplitude (*A* ≃ 10 *μ*m) and low frequency (*f* = 50 − 200 Hz) vibrations. Specifically, it was observed that vibrations cause a drop in the resistive force up to a critical depth. Indeed, for large penetration depths, the resistive force follows a trend similar to the example without vibrations, but shifted downwards. It is qualitatively noted that the critical immersion depth at which the resistive transition occurs increases with vibration acceleration *γ*. By defining this critical depth *z*_*c*_, which maximizes the relative difference (*F*_*γ*_ − *F*_*γ* = 0_)/*F*_*γ* = 0_, the increase can be observed as a function of the vibration acceleration as shown in Fig 3a.

Above the critical depth *z*_*c*_, the tendencies in vertical force in vibrating and non-vibrating experiments are shifted (Fig 2a and 2b). The shift in vertical position *z*_*s*_ between the force and the non-vibrating reference case has been measured and is reported in Fig 3b as a function of the acceleration of vibrations. Similar to the critical depth, the vertical shift increases with *γ* but under a different scaling law. In order to study the origin of this shift, an experiment was carried out where the vibrations of the intruder were stopped during immersion. The result of this experiment is shown in Fig 2c for a cylinder of 8 mm in diameter with a rounded tip entering a granular media made of glass beads of 250 *μ*m in diameter. It is noted that, in agreement with previous observations, the resistive force is reduced in the presence of vibrations. However, when vibrations are stopped (as indicated by the vertical dashed dark line) the resistive force suddenly increases, and finally reaches values similar to those measured for cases without vibrations. This experiment highlights the non-permanent nature of the changes caused by intruder vibrations.

**Fig 4:**
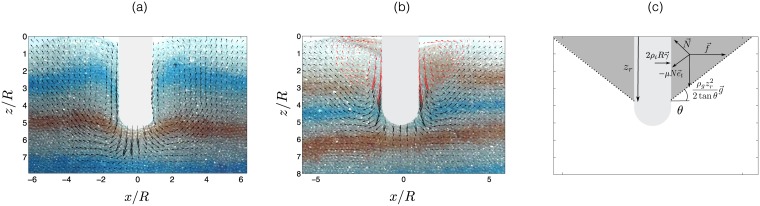
Mean velocity field of grains induced by the intrusion of a flat plate with a cylindrical tip entering at a speed of *U* = 1 mm/s up to a normalized depth of *z*/*R* = 5.2: (a) without vibrations (*γ* = 0m/s^2^) and (b) with vibrations (*γ* = 22m/s^2^). An arrow of unity in length corresponds to a velocity of 1.3 mm/s. The velocity fields are determined by the correlation method used by Hamm *et al.* [9] with an average time of 400 ms. The white zone represents the intruder position where the flow cannot be determined. In (b), the recirculation zones are highlighted with red arrows. (c) Diagram of the forces applied to the granular materials surrounding the intruder.

The effect of intruder vibrations on the surrounding granular media was visualized in a setup similar to the one presented in Fig 1 but with a symmetry invariance along one direction. Recording the motion of grains from the side view and using image correlation techniques similar to Hamm *et al.* [9], it was possible to infer the mean velocity of field of grains induced by intruder motion. Fig 4a and 4b present results for both the vibrating and non-vibrating scenarios. The result from the velocity fields showed an average time of 400 ms, which is considerably longer than the 10 ms period of vibrations. It is noted that in the case without vibrations (Fig 4a), the motion of the tip of the intruder induces grain displacements along a curve which connects the tip of the intruder to the free surface. This curve reflects the path of force chains that develop in the granular media, as previously reported by Hamm *et al.* [9]. In presence of mechanical vibrations, the flow field of the granular material around the finger is modified (Fig 4b). Indeed, grains in contact with intruder walls exhibit velocities exceeding the speed of the intruder (up to a factor of two). This is due to the collision of grains with the surface of the intruder, which vibrates at a typical velocity of *γ*/2*πf* ∼ 4 cm/s. Moreover, Fig 4b shows two recirculating zones at the surface of the granular bed reaching a depth of *z*/*R* ≃ 2.5 (highlighted by red arrows in the figure). This depth corresponds approximatively to the critical depth *z*_*c*_, reported previously for the corresponding vibration acceleration (*γ* = 22m/s^2^). Thus, it is inferred that the vibrations induce a change in the force chains network in a superficial region, which leads to a drop in the resistive force. Furthermore, it is apparent that above the critical depth and at a distance from the intruder, the displacement field is similar that observed in the case without vibrations. This fact explains why above the *z*_*c*_, the resistive force adopts a similar trend to the case without vibrations (Fig 2a and 2b).

## Discussion

**Fig 5:**
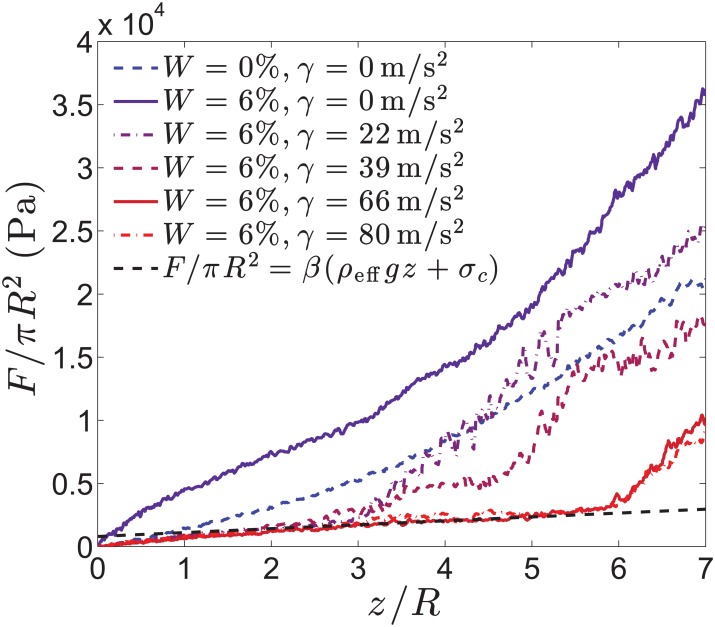
Resistive pressure *F*/*πR*^2^ as a function of the normalized depth *z*/*R* for a vertical penetration (of a cylindrical intruder, *R* = 5 mm, of rounded tip) into a wet granular media at varied accelerations. The media is composed of granular beads of 250 *μ*m in diameter with added interstitial water (water-volume fraction *W* = 6%). For reference, penetration force in a dry material (*W* = 0%) in the absence of vibration is indicated by the dashed blue line. The dark dashed line indicates the best fit of experimental data, within the vibration range that reduces resistance, using the law *F*/*πR*^2^ ≈ *β*(*ρ*_eff_ *gz* + *σ*_*c*_) where *σ*_*c*_ represents the media cohesion. The best fit of experimental data is obtained for *β* = 4 and *σ*_*c*_ = 200 Pa.

The trends observed during this investigation can be rationalized through scaling arguments. First, it is observed that during vibrations and for *z* < *z*_*c*_, all the curves in Fig 2a and 2b collapse on a single one. This shows that resistance is caused by pressure imposed by the granular gas at the tip of the intruder. Consistently, the resistive force can be estimated as *F*/*πR*^2^ = *βρ*_eff_ *gz*, where *ρ*_eff_ is the effective density of the granular gas, *g* is the gravitational acceleration, *z* the depth of the tip and *β* is a numeric constant, of order 1, accounting for dissipation and geometry of the tip. Considering that the effective density is estimated by *ρ*_eff_ ≃ *ρφ* and that compaction is equal to the random close packing (*φ* = 0.63), the previous law is plotted with dashed lines in Fig 2a and 2b and provides a reasonable agreement with experiments for *z* < *z*_*c*_. It is concluded that vibrations create a similar situation to the quasi-static entry of an intruder into a fluid, up to a critical depth *z*_*c*_. However, a clear effect is caused by the geometry of the intruder tip, as intruders with rounded tips (*β* = 2.6) have a more pronounced effect with respect to intruders with conical tips (*β* = 1.1). This trend suggests that round tip intruders are less capable of fluidizing granular compact in contact with the penetrating tip, which leads to some residual vertical force chains. In turn, conical tips can dislodge grains more easily, offering much less probability of pushing grains downward, thus considerably decreasing the probability of vertical force chain formation. Experiments performed in cohesive materials, prepared by increasing the water content of the same particles used for dry tests, present similar trends regarding the reduction of the penetration force under vibration (Fig 5, Supporting Information). However, *F* vs *z* curves are shifted upward giving values comparables with the cohesion, *σ*_*c*_, suggesting that the force law can be generalized as, *F*/2*πR*^2^ ≈ *β*(*ρ*_eff_ *gz* + *σ*_*c*_).

Secondly, the critical depth *z*_*c*_ where the transition on the resistive force occurs is considered. According to the work of Hill *et al.* [6], the observed force from the intruder penetration results mainly due to the stress applied at the tip. It was estimated that the advancing surface undergoes granular stress from the intruder at the depth *z*, scales as *ρ*_*g*_ *gR*^−0.4^ *z*^1.4^ where *ρ*_*g*_ is the density of the granular media. Integrating this stress over the projected surface of the intruder, the total observed force scales as *ρ*_*g*_ *gR*^1.6^ *z*^1.4^. In order to break the force chains, which exist in the granular media and reach a fluidized state, the lateral force induced by the acceleration of the intruder *Mγ* ∼ *ρ*_*i*_ *R*^2^ *Lγ* (*M* being the mass of the intruder, *ρ*_*i*_ its density and *L* its length) and which is transmitted vertically by the tip, must exceed *ρ*_*g*_ *gR*^1.6^ *z*^1.4^. The following scaling law is observed for the critical depth: *z*_*c*_/*R* ∼ (*ρ*_*i*_/*ρ*_*g*_ *L*/*Rγ*/*g*)^0.71^. This is compatible with the data for a range of experimental parameters (Fig 3a, black solid line).

These results indicate that increased vibration acceleration leads to a more pronounced drop in resistive force. Thus, it may be assumed that in order to ease the immersion of an intruder into a non-cohesive material it is possible to use mechanical vibrations of different frequencies, such as ultrasonic vibrations. Indeed, it is known that the typical acceleration generated by ultrasonic transducers is about *γ* ∼ 10^4^ *g* [31]. It is expected that such vibrations allow entry into a granular media with a very low resistance for a penetrating depth up to ten thousand times the transversal size of the intruder. Such an investigation leaves scope for future studies.

However, doubts remain over the relevance of the energy injected into the system as a control parameter for the process of intruder penetration. Indeed, it was recently shown that the difference between a static and dynamic friction can be smoothed out if a small amount of energy is injected into a granular system, and that the main control parameter is the ratio of the injected energy with respect to gravitational energy, at the scale of the roughness size of the surfaces involved [32]. For the current study, the power required to penetrate the media at speed *U* writes, *FU*, which when balanced with the injected power, *ρ*_*i*_ *R*^2^ *LγAω*, leads to *z*_*c*_/*R* ∼ (*ρ*_*i*_/*ρ*_*g*_ *L*/*Rγ*/*g*)^0.71^(*Aω*/*U*)^0.71^. Since the ratio *Aω*/*U* was varied at constant *γ* through the variation of *U*, without significant effect on *z*_*c*_/*R*, it can be concluded that the relevant control parameter is the intruder lateral acceleration, and not the injected energy. Although, the presence of a regime where friction acting on the intruder would be diminished through the input of a small amount of energy cannot be ruled out. In addition, the fact that the penetration force is not a function of particle diameter (for *d* ≪ *R*) indicates that the intruder acceleration is the only relevant parameter for force reduction. Accelerations in the range of a few *g* can be easily achieved at micron size amplitudes and relatively low frequencies. However, micron-size amplitudes may be too small to produce a significant reduction effect for large and heavy particles. As a general rule, the vibration amplitude must be large enough to produce frictional forces demobilization between grains. In our case, for the range of forces applied, grains behave as rigid spheres, leaving the size of asperities (ranging from 10nm to 1000nm) as the only length scale of the contacts. Therefore, vibration amplitudes in the range of 10 *μ*m are larger than this scale and sufficient to ensure friction demobilization. In general, the relative dilation (or shear) amplitude necessary to demobilize friction at grains contacts may depend on several factors, including contact elasticity. For relatively soft particles or at a greater confinement pressure, the typical elastic deformation at the contact is a more relevant scale of vibration amplitude.

Thirdly, the depth of the recirculating zone due to vibrations is considered (Fig 4b) may allow for the optimization of the penetration process. For simplicity, a two-dimensional description of the problem is considered, which is expected to be valid for recirculation zones smaller than the intruder diameter. In this approximation, the linear mass of grains mobilized laterally by the intruder during an oscillation is *ρ*_*g*_ *z*^2^/2 tan *θ*, where *z* is the intruder depth at which the recirculation begins, and *θ* is the angle of wedge measured relative to the horizontal direction and depicted in Fig 4c. The force balance on the center of mass of the wedge includes a friction force, $\mu N{\hat{e}}_{t}$, with a friction coefficient *μ* and a normal reaction *N*, that is tangent to the direction of the lower border of the wedge, ${\hat{e}}_{t}$. This yields to the expression of the lateral force per unit length *f*, as *f* = *ρ*_*g*_ *z*^2^ *g*(1 + *μ*/tan *θ*)/2(1 − *μ* tan *θ*) [33]. For a given depth, the minimum lateral force necessary to overcome friction and mobilize the wedge is $f_{m}=\rho_{g}z^{2}g/(2(\sqrt{\mu^{2}+1}-\mu {)}^{2})$ for an angle *θ*_*m*_ which verifies $\text{tan }\theta_{m}=\sqrt{\mu^{2}-1}-\mu$. Through balancing this minimal lateral force with the force resulting from the intruder acceleration 2*ρ*_*i*_ *Rzγ*, it can be determined that the depth of the recirculating zone *z*_*r*_ is given by $z_{r}/R=2\;(\sqrt{\mu^{2}+1}-\mu {)}^{2}\;(\rho_{i}/\rho_{g})\;\gamma /g$. Consequently, the depth of the recirculating zone is scaled with the vibrations acceleration as *z*_*r*_/*R* ∼ (*γ*/*g*)^1^, contrasting to the critical depth *z*_*c*_ which scales as *z*_*c*_/*R* ∼ (*γ*/*g*)^0.71^. This may explain the presence of a transition between the regime in which the materials surrounding the intruder is fluidized, and the case where the intruder is almost, but not completely, pinned into the granular material. The main feature of this intermediate regime is that, for *z* > *z*_*c*_, intruder force does not attain the potential value under the absence of vibrations (Fig 2a and 2b). Thus, the existence of the recirculation zone remains the most probable explanation of why force curves appear shifted with respect to those in the absence of vibration for *z* > *z*_*c*_. If we define a penetration *z*_*s*_ as the shift necessary to superimpose each curve force with the reference curve without vibrations in the range *z* > *z*_*c*_, we obtain data of Fig 3b, validating that *z*_*s*_/*R* scales linearly with (*γ*/*g*), which is consistent with the previous prediction. Thus, the force shift occurs due to existence of an effective penetration depth defined as *z* − *z*_*s*_, and arising from the fact that grains located in the recirculation zone, i.e., above *z*_*s*_, do not impose a force on the intruder.

Finally, this investigation raises the question of how force chains in granular media can be broken, reducing the resistance of these materials toward penetration. It can be presumed that while entering granular media it is appropriate to impose oscillatory rotations of small amplitudes or mechanical pulses spaced in time on the intruder. The possible use of these strategies in nature by animals and plant roots (at a very different time scale) is a fascinating question that remains to be explored. Moreover, the potential for improvement of diggers and robots through the introduction of small amplitude vibrations is the principal result of this study.

## Supporting information

S1 Fig This section presents a brief overview of the effects that media cohesion and intruder vibration have on the penetration force. In order to adjust the cohesion and simultaneously carry out a direct comparison of the penetration force with that of a dry granular sample, a mass of water was added to the granular media. The sample was prepared as described in Fall *et al.* [34]. The results are presented in Fig 5. In both dry and wet cases, at the same intruder acceleration, there is a significant force reduction induced by vibration. However, resistive force increases with cohesion compared with the values obtained at null cohesion. It is noted that interstitial water creates capillary bridges between grains and thus induces a cohesive force, increasing the confinement pressure over all depths. In addition, a decrease in resistive force is observed up to a certain depth, similar as in the dry granular media. These results indicate that the scenario leading to force reduction in wet granular media may not significantly differ from that in dry media, however the role of water bridges as a lubricant agent deserves further investigation.
(EPS)

S1 Spreadsheet Raw data of the penetration reduced force *F*/(*πR*^2^) as a function of the reduced depth *z*/*R* for various vibration acceleration *γ* and different intruder geometry (radius *R* and tip shape). In addition, the values of the normalized critical depths *z*_*c*_/*R* and *z*_*s*_/*R* are provided as a function of the reduced acceleration *γ*/*g*.
(XLS)
